# Measurement of serum tenascin-X in patients with congenital adrenal hyperplasia at risk for Ehlers–Danlos contiguous gene deletion syndrome CAH-X

**DOI:** 10.1186/s13104-019-4753-7

**Published:** 2019-10-30

**Authors:** Vipula Kolli, Hannah Kim, Hamsini Rao, Qizong Lao, Alison Gaynor, Joshua D. Milner, Deborah P. Merke

**Affiliations:** 1 National Institutes of Health Clinical Center, 10 Center Drive, Building 10, Room 1-2740, Bethesda, MD 20892-1932 USA; 2 The Eunice Kennedy Shriver National Institute of Child Health and Human Development, Bethesda, USA; 3 National Institute of Allergy and Infectious Diseases, Bethesda, MD 20892 USA

**Keywords:** Congenital adrenal hyperplasia, Ehlers–Danlos syndrome, Tenascin-X, *CYP21A2*, *TNXB*

## Abstract

**Objective:**

Approximately 10% of patients with congenital adrenal hyperplasia (CAH) due to 21-hydroxylase deficiency carry a mutation that disrupts *CYP21A2* and the flanking *TNXB* gene resulting in CAH-X, a contiguous gene deletion syndrome. *TNXB* encodes tenascin-X (TNX), an extracellular matrix glycoprotein that plays an important role in collagen organization. *TNXB* impairment is associated with Ehlers–Danlos syndrome. Symptoms include joint hypermobility, hernias and cardiac defects. We measured serum TNX using an antibody targeting the amino-terminal of the TNX protein in 161 subjects, including extensively genotyped and phenotyped CAH patients, their relatives, and healthy controls.

**Results:**

We evaluated the potential of serum TNX as a screening tool for CAH-X. CAH-X patients, especially haploinsufficient patients carrying the *TNXA*-*TNXB* chimeric gene CAH-X-CH-1 showed reduced TNX levels compared to controls (*P *< 0.05). TNX levels were similar in all subjects carrying a *TNXB* mutation. However, CAH patients who did not harbor a *TNXB* mutation also had reduced TNX compared to controls (*P* < 0.001). Thus, measuring serum TNX is not an effective screen for CAH-X amongst patients with CAH. *TNXB* genotyping is recommended for CAH patients who have symptoms of a connective tissue disorder. Epigenetic factors that influence TNX expression require further study.

## Introduction

Mutations of the *CYP21A2* gene encoding 21-hydroxylase (21-OH) cause congenital adrenal hyperplasia (CAH), an autosomal recessive disorder of steroidogenesis. Deficiency of 21-OH leads to insufficient cortisol and aldosterone with excess androgen production and is classified as classic or non-classic depending on the degree of enzyme impairment [1]. The classic form is potentially life-threatening, estimated to affect approximately 1 in 15,000 newborns and part of the mandatory neonatal screen in all 50 U.S. States and in over 40 countries [2]. The mild nonclassic form is one of the most common autosomal recessive disorders, occurring in 1 in 200 to 1000 Caucasians of mixed ethnicity [3].

The *TNXB* gene encoding tenascin-X, an extracellular matrix protein, was first identified during studies of *CYP21A2* due to overlapping genetic sequences [4, 5]. TNX, a large glycoprotein, is highly expressed in connective tissue and plays an important role in collagen fibrillogenesis and matrix maturation [6]. *TNXB* mutations affecting both alleles cause a severe autosomal recessive form of Ehlers–Danlos syndrome (EDS), a connective tissue disorder characterized by hypermobile joints and tissue fragility [7] and a subset of those with the hypermobility type have been found to have *TNXB* haploinsufficiency with reduced serum levels of TNX [8]. Approximately 10% of patients with CAH carry a 30-kb *CYP21A2* deletion extending into the flanking *TNXB* gene on at least one allele causing a contiguous gene deletion syndrome termed CAH-X [9, 10]. Patients with CAH-X suffer from CAH and hypermobility type EDS [9, 10]. Most CAH-X diagnoses result from two types of *TNXA/TNXB* chimeric genes: CAH-X CH-1 contains a 120 bp deletion spanning exon and intron 35 resulting in haploinsufficiency. CAH-X CH-2 arises from a missense mutation c.12174C>G (p.Cys4058Trp) in exon 40, which likely disrupts TNX function but not expression [10, 11]. Patients with CAH-X display a range of connective tissue abnormalities including joint hypermobility and subluxations, chronic arthralgia, hernias and cardiac defects [9, 10, 12].

In this study, we measured serum TNX in a large cohort of patients with CAH who had undergone comprehensive genetic analysis, their relatives who carry *TNXB* mutations, and healthy controls. We evaluated the potential use of serum TNX as a biomarker of CAH-X. We also examined the association between TNX levels and *TNXB* genotype.

## Main text

### Study population

Subjects were enrolled in the Natural History study at the National Institutes of Health (NIH) Clinical Center in Bethesda, MD, USA (clinical trials no. NCT00250159). Blood samples were obtained from 119 subjects with known *CYP21A2* and *TNXB* genotypes, age 2 to 74 years. We evaluated 70 CAH patients without *TNXB* impairment, 33 CAH-X patients, and 16 carriers of CAH-X. Most of the CAH patients were receiving glucocorticoid replacement therapy (17 patients on dexamethasone, 21 prednisone, 48 hydrocortisone, 15 unmedicated (14 with nonclassic CAH), and 1 unknown; average hydrocortisone equivalency dose for those receiving treatment 23.2 ± 17.3 mg/day). Patients with CAH-X and CAH only were receiving comparable glucocorticoid doses (23.3 ± 16.0 *vs*. 23.2 mg ± 17.9 mg/day). All patients were examined for joint and skin abnormalities prior to genotyping. The Beighton 9-point scale was used to score joint hypermobility by one physician (DPM) and generalized hypermobility was defined according to the 2017 International Classification of the EDS [13]. Skin hyperextensibility was evaluated by stretching the volar skin of the forearm. Measurement of more than 1.5 cm was considered hyperextensible. Signs and symptoms of a connective tissue dysplasia were collected from medical record review and during interview and review of systems at each visit. Our control population consisted of 42 age- and sex-matched healthy subjects who donated blood to the NIH blood bank or enrolled in clinical trials no. NCT01164241 as healthy controls.

### Enzyme-linked immunosorbent assay

Serum samples were isolated by centrifuging blood at 3000 rpm for 15 min. TNX was detected in the serum with a double-antibody sandwich enzyme-linked immunosorbent assay targeting the amino-terminal amino acids of TNX according to the manufacturer’s instructions (Cloud-Clone Corp., Katy, TX, USA). Experiments were conducted in duplicates. Protein concentrations were obtained by comparing optical density (OD) values of the samples to the OD standard curve generated using known protein concentrations.

### Statistical analysis

Statistical analyses were performed using KaleidaGraph Version 4.1 (Synergy Software, PA). The nonparametric Kruskal–Wallis test was applied to analyze differences among and between the groups. Fisher’s Least Significant Difference test was employed when comparing two groups. Data are represented as median (interquartile range). *P*-value of ≤ 0.05 was considered significant. The Spearman’s Rank Correlation Coefficient was used to evaluate the association between the TNX concentrations and age.

## Results

### Clinical findings of TNX deficiency among CAH-X patients

As expected, patients with CAH-X had clinical symptoms of a connective tissue disorder, such as generalized hypermobility, subluxations, chronic arthralgia and hyperextensible skin, than CAH patients without a *TNXB* mutation (Table 1). CAH-X CH-2 caused a more severe phenotype than CAH-X CH-1, with increased joint and skin manifestations, while biallelic CAH-X patients had the most severe phenotype with significant joint hypermobility and skin hyperextensibility. Relatives without CAH but with heterozygous *TNXB* mutations had milder hypermobile EDS phenotype compared to CAH-X patients (Table 1).

**Table 1:** Clinical characteristics of a cohort of CAH-X and CAH patients and carriers

	Monoallelic CAH-X CH-1 (*n *= 16)^a^	Monoallelic CAH-X CH-2 (*n *= 13)	Biallelic CAH-X (*n *= 3)	CAH-X carrier Relatives (*n *= 16)	CAH only (*n *= 70)
Age, years (range)	23.1 ± 10.5 (4–38)	23.2 ± 15.5 (5–43)	21.3 ± 7.6 (14–29)	49.4 ± 16.2 (20–74)	25.7 ± 14.9 (2–68)
Female	8 (50.0)	7 (53.8)	0 (0)	10 (62.5)	43 (61.4)
Generalized hypermobility^b^	6 (42.9) (n = 14)^e^	6 (46.2)	3 (100)	3 (20) (n = 15)^e^	6 (12.2) (n = 49)^e^
Subluxations	6 (40.0)	5 (38.5)	2 (66.7)	1(6.7) (n = 15)^e^	12 (17.1)
Chronic arthralgia	5 (31.3)	4 (30.8)	1 (33.3)	7 (43.8)	2 (2.8)
Chronic tendonitis	2 (12.5)	4 (30.8)	0 (0)	3 (20) (n = 15)^e^	2 (2.8)
Hyperextensible skin	2 (12.5)	7 (53.8)	3 (100)	0 (0) (n = 15)^e^	0 (0)
Wide scars	3 (18.8)	2 (15.4)	2 (66.7)	0 (0) (n = 15)^e^	0 (0)
Easy bruising	5 (31.3)	4 (30.8)	3 (100)	1 (6.7) (n = 15)^e^	0 (0)
Poor wound healing	0 (0)	0 (0)	1 (33.3)	0 (7.1) (n = 15)^e^	0 (0)
Gastrointestinal disorder^c^	1 (6.3)	4 (30.8)	1 (33.3)	0 (0) (n = 15)^e^	4 (5.7)
Hernia or rectal prolapse	0 (0)	3 (23.1)	2 (66.7)	1 (6.7) (n = 15)^e^	1 (1.4)
Congenital cardiac defect^d^	3 (18.8)	3 (23.1)	0 (0)	3 (20.0) (n = 15)^e^	0 (0)
Cardiac chamber enlargement	2 (14.3) (n = 14)^e^	0 (0) (n = 10)^e^	2 (66.7)	4 (26.7) (n = 15)^e^	1 (2.9) (n = 35)^e^
Enlarged aortic root	0 (0) (n = 14)^e^	2 (20) (n = 10)^e^	0 (0)	2 (13.3) (n = 15)^e^	0 (0) (n = 34)^e^

Values are numbers with percentages in parentheses or mean ± standard deviation

^a^One patient omitted due to lack of clinical data

^b^Generalized hypermobility defined as a Beighton score of 4 of 9 or above for those > 50 years of age, 5 of 9 or above for post pubertal adolescents and adults, and 6 of 9 or above for children [13]

^c^Gastrointestinal chronic disorder included irritable bowel syndrome and gastroesophageal reflux disease

^d^Cardiac congenital disorder included left ventricular diverticulum, patent foramen ovale, or a structural valve abnormality

^e^Values adjusted for missing data

### Measuring tenascin-X serum levels in patients with CAH and CAH-X

**Fig. 1:**
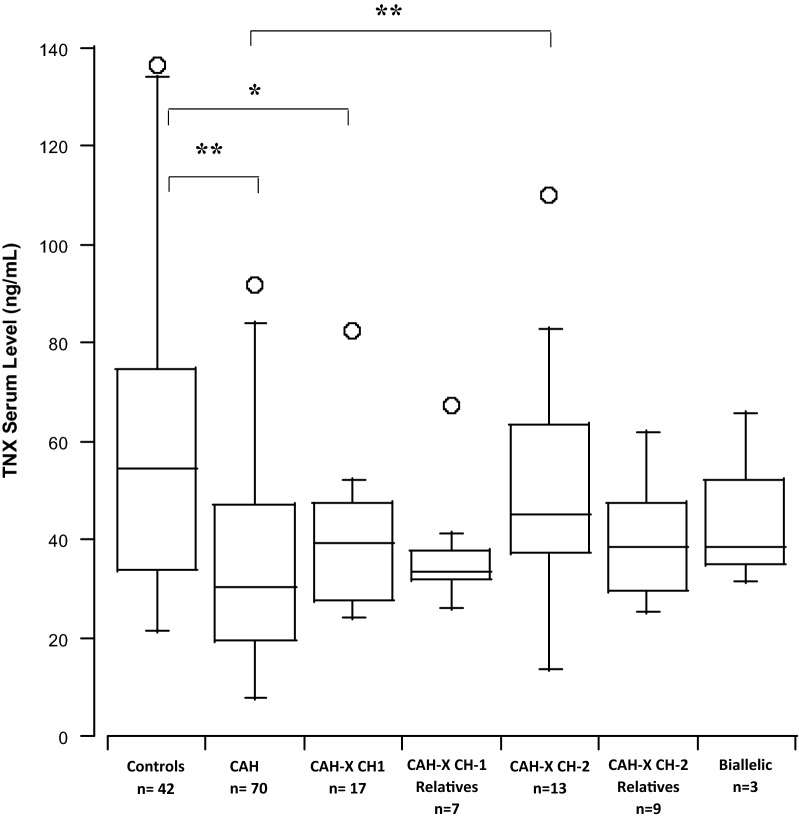
Serum tenascin-X in healthy volunteers, patients with CAH and different CAH-X patient groups. Box plots represent the median (interquartile range) of absolute serum TNX concentrations. *P < 0.05, **P < 0.001, as determined by Kruskal–Wallis test. Comparisons between other groups were not significant

**Fig. 2:**
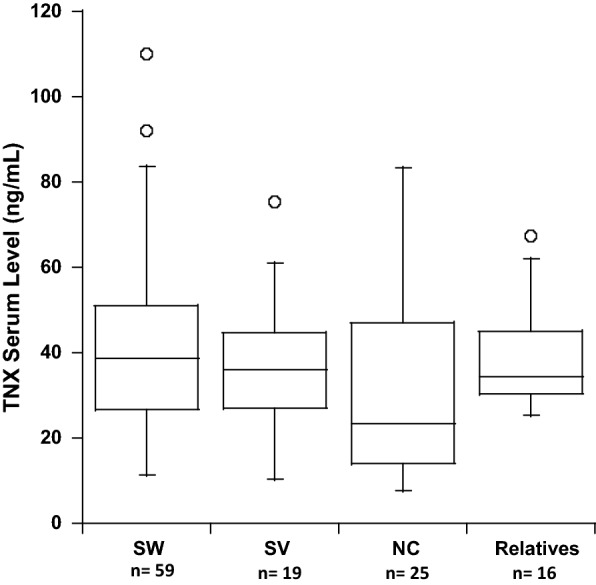
Serum tenascin-X according to the CAH phenotype. Box plots represent the median (interquartile range) of absolute serum TNX concentrations. SW: salt-walting, SV: simple virilizing, NC: non-classic

TNX was readily detected in the serum of all subjects. Compared to controls, TNX levels were significantly reduced in patients with CAH carrying a CAH-X CH-1 chimera (*P *< 0.01) (Fig. 1). However, despite differences in phenotype, CAH patients with monoallelic CAH-X CH-1, monoallelic CAH-X CH-2 and biallelic CAH-X had similar serum TNX levels (*P *= 0.35; Fig. 1). Surprisingly, TNX levels were reduced in CAH patients without *TNXB* impairment by approximately 40% compared to healthy controls (*P* < 0.0001; Fig. 1). Levels did not differ according to CAH phenotype (*P *= 0.07; Fig. 2), sex (males: median 39.3 ng/mL, IQR 30.1 to 57.1; females: median 34.7 ng/mL, IQR 23.6 to 57.5; *P* = 0.43) or age (r_s_ = 0.178).

## Discussion

Our study is the first to evaluate the potential use of serum TNX as a biomarker of CAH-X in patients with 21-OHD CAH. Due to the *CYP21A2*-*TNXB* gene overlap, patients with 21OHD CAH are at risk for hypermobility type EDS due to TNX impairment. Patients with CAH-X have variable joint, skin and cardiac findings [9, 10, 12]. Screening for CAH-X in CAH patients and their family members is essential for implementing preventative care. We found a similar reduction of TNX levels in all CAH patients compared to controls, irrespective of the presence or absence of *TNXB* mutations. Thus, measuring serum TNX in patients with CAH is not a useful screening tool for CAH-X, and we recommend genotyping for diagnostic purposes.

EDS is a heterogeneous group of connective tissue disorders and measuring serum TNX has been used to screen for a TNX-related etiology in patients with EDS. Schalkwijk et al. reported that serum TNX was undetectable in 5 patients with EDS who were subsequently identified as having autosomal recessive *TNXB* mutations. Further studies had similar results: low serum TNX was found to be associated with autosomal recessive EDS due to complete loss of tenascin-X [14, 15]; and reduced TNX was observed in females with hypermobility type EDS due to *TNXB* haploinsufficiency [8]. Our results mostly confirm these findings; we found that CAH-X CH-1, the mutation identified in these prior EDS reports, was associated with lower serum TNX levels than healthy controls. However, the wide range of values obtained in both affected and unaffected subjects argues against the utility of measuring serum TNX.

Our study failed to find differences in TNX levels between different CAH-X chimeras. The CAH-X chimera types identified to date retain specific *TNXA* pseudogene variants. We previously described decreased TNX expression in CAH-X CH-1 dermal fibroblasts, but unchanged TNX expression in CAH-X CH-2, suggesting haploinsufficient and dominant–negative mechanisms respectively [9, 10]. The c.12174C >G (p.Cys4058Trp) mutation associated with CAH-X CH-2 was found to disrupt TNX function but not affect protein expression. Therefore, we predicted that subjects carrying CAH-X CH-1 would have lower serum TNX levels than subjects carrying CAH-X CH-2. Also, because patients with biallelic CAH-X have a more severe EDS phenotype than patients with monoallelic CAH-X [11], we expected lower TNX levels. Surprisingly, no differences were found amongst these CAH-X groups. All biallelic CAH-X patients had at least one CAH-X CH-2 allele, possibly contributing to this finding.

Unexpectedly, we found a similar reduction of serum TNX in CAH patients with and without a *TNXB* mutation. One possibility for reduction of TNX in CAH patients is the negative regulation of TNX by glucocorticoid medication administered to CAH patients [16, 17]. Recently Yamada et al. measured serum TNX using nano-liquid chromatography tandem mass spectrometry in patients with hypermobility type EDS and reported a decrease in measured serum TNX levels in half of the tested 17 patients. *TNXB* mutations were not found in these EDS patients with decreased serum TNX concentration, suggesting that there are other factors influencing TNX expression [18]. Other evidence exists supporting the concept of unknown epigenetic effects on TNX expression. CAH-X patients reportedly have a more severe EDS phenotype than their relatives who carry the same *TNXA/TNXB* chimera [9]. Affected relatives may have no manifestations or display mild symptoms of hypermobility type EDS [8, 12]. This variable and less severe phenotype suggests an underlying influence of a CAH-related factor on TNX and the EDS phenotype.

Studies to date measuring serum TNX as a screen for a TNX-related etiology in EDS patients have used an ELISA targeting the carboxyl-terminal side of the TNX protein, where the CAH-X variants are located [7, 8, 19]. At the time of these studies, only the CAH-X CH-1 mutation had been identified. The antibody used in our study was raised against human EGFL 15 to EGFL 19 domains, the amino-terminal part of TNX. Although the difference in antibodies might explain differences in results, an amino-terminal recognizing antibody might recognize the full length or fragments of the TNX protein. Serum levels were expected to reflect the expression or post-translational process of full length TNXB protein affected by the CAH-X variants. Egging et al. reported the presence of different TNX fragments using a polyclonal antibody recognizing the carboxyl-terminal portion of TNX, and another polyclonal antibody raised against the fibronectin type III 29-30 domains of TNX. Multiple TNX fragments were found in the serum of unaffected but not in TNX deficient patients. Thus, we hypothesized that in serum of an affected CAH X-CH1 (haploinsufficient) patient, the total amount of TNX protein, including fragments, might be decreased. In fact, we did find this to be true, however, this finding was not specific to CAH-X CH-1. Alternative antibodies were not available for our use. We therefore recommend *TNXB* genotyping for diagnostic purposes for patients with CAH who have 2 or more signs and symptoms of a connective tissue disorder, such as multiple subluxations, generalized hypermobility, hyperextensible skin and hernias [20].

### Limitations

The lack of significant differences between the CAH-X groups and between CAH-X CH-1 and CAH patients highlights the challenges of using an ELISA assay to screen for TNX deficiency. In addition to possible epigenetic effects on TNX expression, sample quality variations caused by collection or separation methods, operating systems, timing or even the conditions of subjects might have played a role in the wide variability of our measurements. Although CAH-X biallelic patients were included in our study, patients with homozygous CAH-X CH-1 were lacking. An antibody raised against the TNX carboxyl-terminal side was not available for comparison purposes. Developing an assay that targets a peptide based on known TNX mutations might improve the specificity and sensitivity of the assay. In general, measuring serum TNX, especially using an antibody targeting the amino-terminal of the TNX, does not appear to be a useful biomarker of EDS due to TNX impairment, and this approach is not a reliable method for screening patients with CAH.

## Data Availability

Not applicable.

## Abbreviations

CAH: congenital adrenal hyperplasia
21-OD: 21-hydroxylase
TNX: tenascin-X
EDS: Ehlers–Danlos syndrome
